# Open housing drives the expression of immune response genes in the nasal mucosa, but not the olfactory bulb

**DOI:** 10.1371/journal.pone.0187192

**Published:** 2017-10-27

**Authors:** Carolin Piotrowski, Vera Lede, Anne Butthof, Nicole Kaiser, Petra G. Hirrlinger, Matthias H. Tschöp, Torsten Schöneberg, Ingo Bechmann

**Affiliations:** 1 Institute of Anatomy, Faculty of Medicine, University of Leipzig, Leipzig, Germany; 2 Rudolf Schönheimer Institute of Biochemistry, Faculty of Medicine, University of Leipzig, Leipzig, Germany; 3 Medical Experimental Center, Faculty of Medicine, University of Leipzig, Leipzig,Germany; 4 Helmholtz Center for Environmental Health, Munich, Germany; Universite de Lyon, FRANCE

## Abstract

Nasal mucosa and olfactory bulb are separated by the cribriform plate which is perforated by olfactory nerves. We have previously demonstrated that the cribriform plate is permissive for T cells and monocytes and that viruses can enter the bulb upon intranasal injection by axonal transportation. Therefore, we hypothesized that nasal mucosa and olfactory bulb are equipped to deal with constant infectious threats. To detect genes involved in this process, we compared gene expression in nasal mucosa and bulb of mice kept under specific pathogen free (SPF) conditions to gene expression of mice kept on non-SPF conditions using RNA deep sequencing. We found massive alterations in the expression of immune-related genes of the nasal mucosa, while the bulb did not respond immunologically. The absence of induction of immune-related genes in the olfactory bulb suggests effective defence mechanisms hindering entrance of environmental pathogens beyond the outer arachnoid layer. The genes detected in this study may include candidates conferring susceptibility to meningitis.

## Introduction

The nasal mucosa is a location of many facultative pathogenic germs, such as Haemophilus, Staphylococcus and Neisseria, which are potential causes of meningitis [1]. Meningitis is an often lethal infectious disease which can affect also children and adolescents without known immune defect. *Neisseria meningitidis* is a commensal resident of the human pharyngeal mucosa [2] where binding of neisserial colony opacity-associated protein adhesins (Opa) to carcinoembryonic antigen-related cell adhesion molecule CEACAM 1 induces an inflammatory response [3]. It has been calculated that less than 1 in 25,000 natural infections in humans lead to invasive meningococcal disease during endemic periods [4] rendering likely genetic variants driving invasive disease as it has been shown for deficiencies in the complement system [5]. Thus, the nasal mucosa is likely to be a site of permanent interaction with infectious agents which by their presence induce the expression of gene products required for successful defence. Knowledge of these genes may be of help to identify alleles conferring risk for the development of certain forms of meningitis.

Here, we reasoned that the respective mRNAs are expressed only at low levels in mice kept under specific pathogen free (SPF) conditions. Allowing commensalism by transferring subgroups of mice from SPF to open housing and subsequent comparison of mRNA expression using next generation deep sequencing should then provide a list of defence genes which become pathogen-induced in nasal mucosa cells or become present due to specific cell invasion into the nasal mucosa. In fact, massive impact of housing on immune status has just been shown [6,7]. Since we have previously shown that olfactory nerves can serve as entrance routes for virus [8] and that the cribriform plate is permissive for cells [9,10], we also tested as to how far the olfactory bulb senses and responds to commensalism. We show that the nasal mucosa—but not the olfactory bulb—exhibits massive changes in gene expression upon being opposed to an open environment.

## Material and methods

### 1. Samples and animal ethics

Animal husbandry was performed in the animal facilities of the Faculty of Medicine, University of Leipzig according to European (Council Directive 86/609/EEC) and German (“Tierschutzgesetz”) guidelines for the welfare of experimental animals and approved by the local authorities (Landesdirektion Sachsen; T69/13; T32/14). Mice were housed in a 12 h/12 h light-dark cycle with access to food and water *ad libitum*. Thirty male C57/Bl6J mice (six weeks old) with SPF status were purchased from Janvier Labs. Animals were kept together in the central breeding facility of the Medical Faculty of the University of Leipzig for one week to allow for acclimatization. Afterwards 15 mice were transferred to a satellite animal facility and kept under non-SPF conditions for additional one or two weeks, respectively. The SPF husbandry had stable overpressure in all rooms, the air was filtered and the air temperature and humidity was stable. The cages were cleaned mechanically every week and rinsed with 80°C water afterwards. Only animal care personnel had access to the rooms and changed into hygienic clothing, breathing protection and gloves previous to entering. Every three years a worm prophylaxis was conducted and there where routine health controls according to FELASA standard protocols. In the satellite facility the animals were kept in a room, freely accessible to all scientists and employees. The cages were washed manually every 14 days and had no filter lid. The room had no air filtering system and no overpressure. No worm prophylaxis was performed and there were no routine health control according to FELASA standard protocols. Furthermore, *mouse hepatitis virus* (MHV) and *syphacia species* was attested in this animal husbandry by using contact sentinel mice.

### 2. Tissue preparation for RNA isolation

Isoflurane (Baxter) was used to anesthetize the mice, which were quickly decapitated afterwards. To avoid differences due to circadian gene expression all animals were prepared approximately at the same daytime (3 pm). The dissecting set and workplace were cleaned with RNaseZap® (Qiagen) to eliminate the RNases. 1.5 ml tubes were cooled in dry ice and filled with RNAlater^TM^ (Qiagen). The nasal dorsum was incised to gain access and isolate the nasal mucosa. To extract the olfactory bulb the skull was opened via a Y-formed cut and carefully detached. The tissue was frozen immediately at -80°C until the RNA preparation was performed.

### 3. Histology and microglial quantification

Mice were perfused with 4% paraformaldehyde (PFA) and fixed overnight with 4% PFA. After decalcifying the mice heads with EDTA (ethylenediaminetetraacetic acid) for two weeks, standard sucrose solutions (10%, 20% and 30%) were applied consecutively. Samples were sectioned with a cryostat. Gram staining and Immunofluorescence staining was performed according to standard protocols. To visualize microglial morphology in the olfactory bulb polyclonal IBA-1 antibody (WAKO, 1:200; second antibody Alexa goat anti rabbit 568, 1:500) and DAPI (4',6-diamidino-2-phenylindole dihydrochloride, 1:10,000) were used. For microglial quantification five pictures at day 0 and day 5 from each husbandry condition were analysed. Activated and ramified microglia were counted with Image J [11] according to Pouchoulen et. al (2015) [12]. Statistics was performed with Microsoft Excel.

### 4. RNA sequencing of olfactory bulb and nasal mucosa

Total RNA from isolated bulb and mucosa was extracted by using the TRI REAGENT™ (Sigma-Aldrich) as described in the manufacturer’s instructions. The quantity of the RNA was measured using a spectrophotometer (Nanodrop ND 1000) and RNA quality of all samples was examined on the Agilent 2100 bioanalyzer using the RNA 6000 Nano Chip (Agilent Technologies, Santa Clara, CA). We only included RNA samples with a RIN value above 8. Indexed cDNA libraries were generated using TruSeq RNA Sample Preparation Kits v2 (Illumina, San Diego, CA, USA) according to the manufacturer’s protocol, constructing libraries with an average size of 300bp as evaluated on the Agilent 2100 bioanalyzer with DNA 1000 Chips. The libraries were sequenced on the Illumina HiSeq 2500, generating 101-bp raw paired-end reads on 4 flow cell lanes (Max Planck Institute of Evolutionary Anthropology, Leipzig). Briefly, after quantification of the libraries using the Library Quantification Kit, Illumina/Universal (KAPABiosystems) according to the instructions of the manufacturer products were used for cluster generation. Library DNA at a concentration of 10 pM was clustered using an Illumina cBot according to the PE_Amp_Lin_Block_Hybv8.0 protocol of the manufacturer. Sequencing was performed using version 3 chemistry and the version 3 flowcell according to the manufacturer’s instructions. Median cluster density was usually about 600,000 clusters per mm^2^ or 80–100 million raw clusters per lane. After intensities call, raw reads were separated according to library indexes allowing up to one mismatch in the index sequence, but requiring that all bases have a quality score above 15 (PHRED-scale). After assigning reads to samples we used an in-house-sequencing pipeline to trim the adapters and remove reads, which were shorter than 60 bp or have more than five bases with a quality score below 15 (PHRED-scale). Reads were mapped to the reference mouse genome (July 2007 NCBI37/mm9) with Ensembl v66 annotations using Tophat 2.0.6. [13,14], which aligns reads using Bowtie2 (version 2.1.0). Mitochondrial reads and reads which did not map uniquely to a genome position were excluded. The transcription level for each gene was obtained by intersecting mapping results with gene annotations using BEDTools IntersectBed [15]. Using DESeq software package [16], differential expression of genes under SPF and non-SPF conditions was examined. Only genes that were expressed at least in half of the animals were included for analyses. Differentially expressed genes (DEGs) with a p-value <0.05 were considered as statistically significant.

### 5. Data analysis

We used tables of all significant differentially expressed genes (DEGs) from each tissue (bulb and mucosa) of the distinct points of time (one and two weeks), which were altered when compared between housing conditions. These sets were subjected to gene ontology (GO) analysis with DAVID Bioinformatics Resources 6.7 [17,18]. With these lists we gained "functional annotation charts" only with DAVID's 3 default GO term libraries. Only GOs with a p-value <0.05 and FDR<0.1 were included into further analysis.

## Results

### 1. Analysis of RNA-Seq data

**Fig 1:**
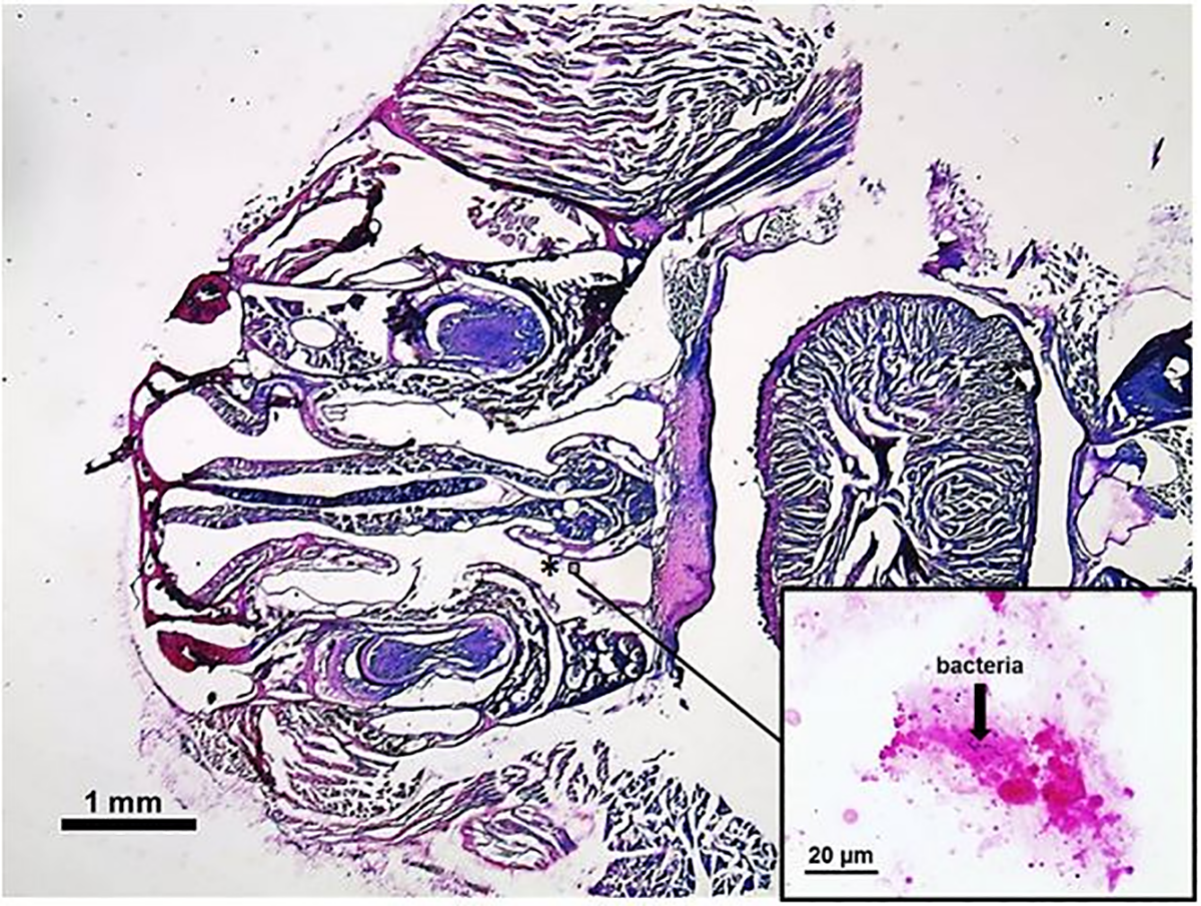
Gram staining of a mouse whole head section. A horizontal section of a mouse head was stained by Gram´s method to show the occurrence of gram positive bacteria at the nasal mucosa (*).

**Fig 2:**
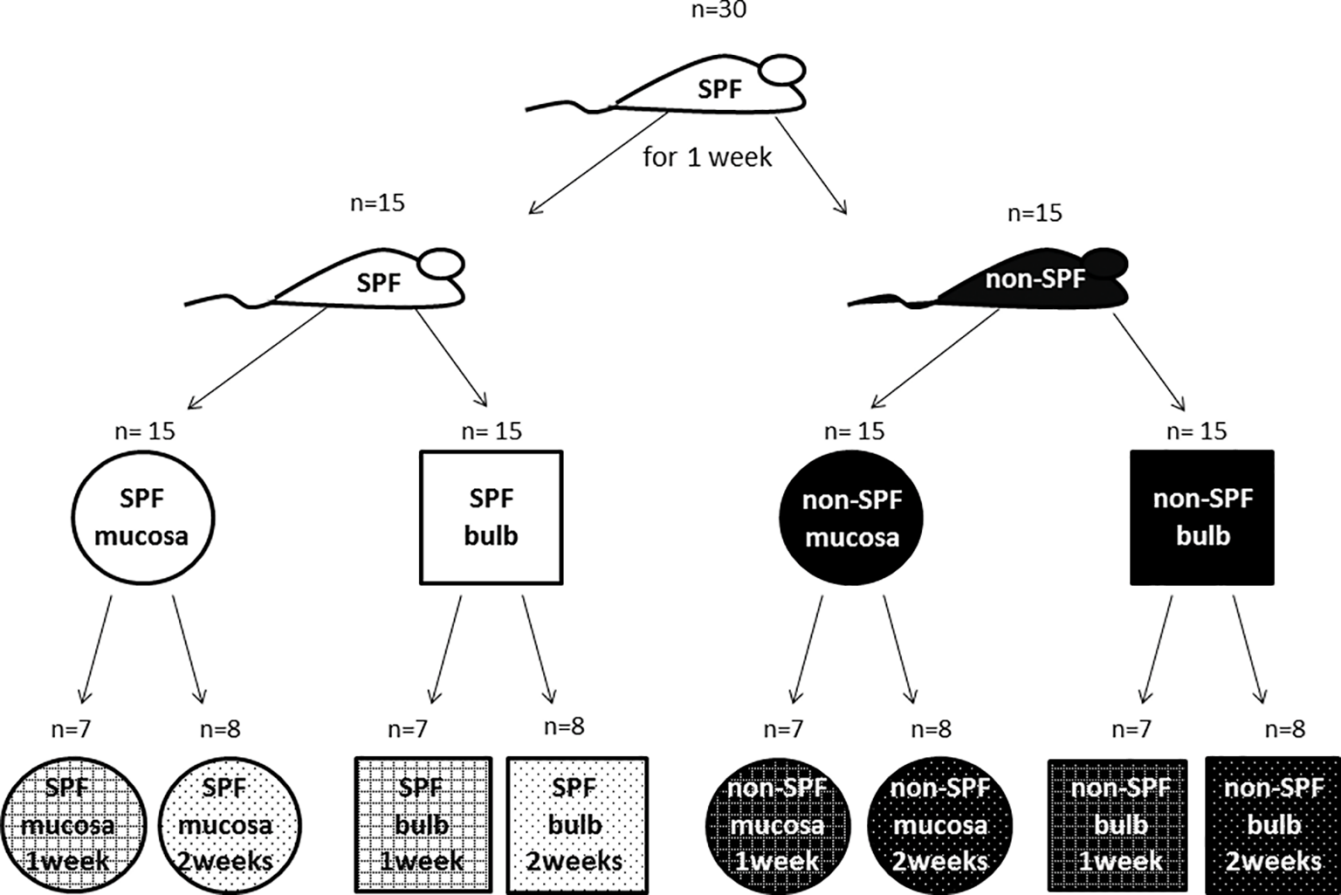
Experimental setup. 30 mice were obtained for this experiment and kept together for one week in a SPF facility for adaptation. Afterwards 15 mice were transferred to a non-SPF environment. After one week of different husbandry conditions (SPF vs. non-SPF) 7 animals of each group were euthanized followed by the removal of the olfactory bulb and the nasal mucosa. The same procedure was performed with the remaining mice after two weeks.

**Fig 3:**
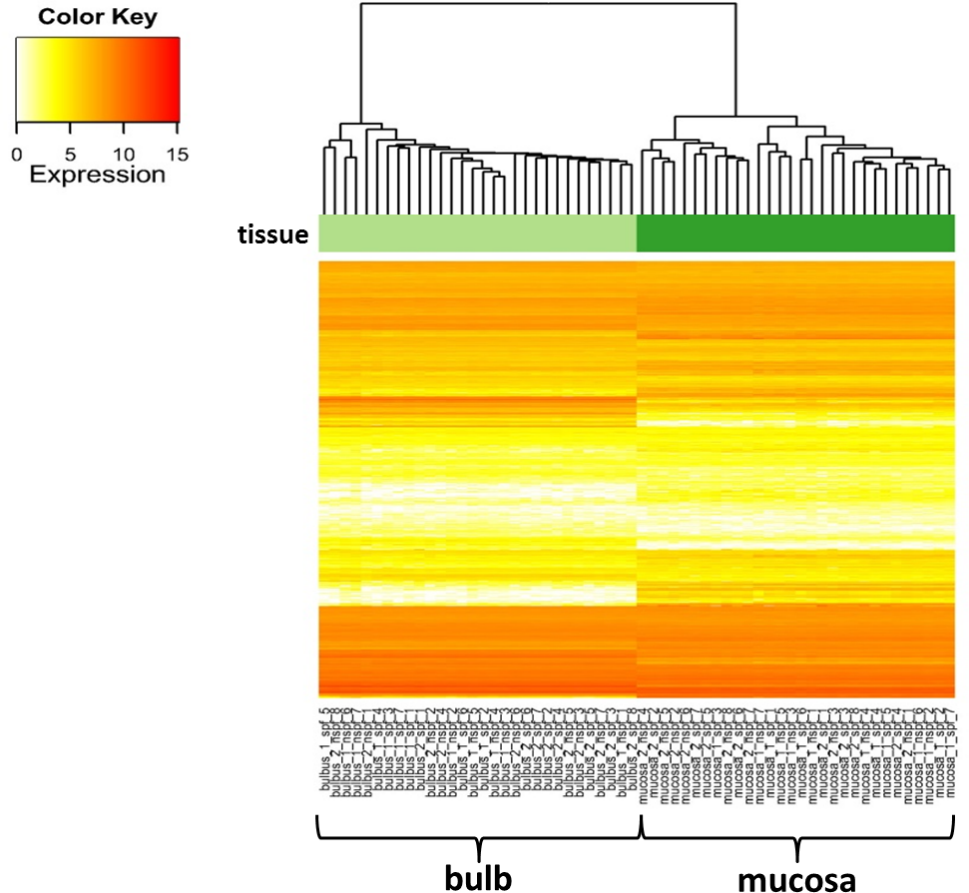
Heatmap illustrating separation of olfactory bulb and nasal mucosa. The X axis exhibits the particular probes of mucosa (e.g. mucosa after 2 weeks of SPF husbandry, probe 1 = "muc_2_spf_1") and olfactory bulb. On the Y axis expression of all mapped genes is displayed. The hierarchical clustering on top shows the actual splitting of the two tissues.

We worked with 60 samples from 30 seven weeks old animals consisting of 30 nasal mucosae (Fig 1) and 30 olfactory bulbs, 15 from SPF and 15 non-SPF animals, respectively. Of these 15 probes, 7 samples were taken from the specific housing condition after one week, 8 samples derived from mice kept for 2 weeks in their environment (Fig 2). Each sample thus had three qualities: the husbandry (SPF or non-SPF), the point of time (1 week or 2 weeks) and the tissue (olfactory bulb or nasal mucosa). Using the DEseq software package for each analysis two of these three factors were constant, e.g. bulb and 1 week. In this example, from the 60 samples each one with the two qualities "bulb" and "1 week" was selected, resulting in 14 probes which differed in the third factor “husbandry” of which 7 derived from SPF and 7 from non-SPF conditions. Comparison of these 7 versus 7 probes resulted in a list of all genes which were differentially expressed after one week. Table 1 shows the obtained mean of raw reads, reads after quality control and of the uniquely mapped reads for all analyses. Altogether, we sustained an average number of 35 million raw reads per sample. After quality control an average of 33.6 million reads remained. We used TopHat to align the reads to the reference mouse genome (July 2007 NCBI37/mm9). Almost 26 million reads were uniquely mapped per sample whereat mitochondrial reads were excluded. To verify the separation of the tissues we created a heatmap of all mapped genes. Fig 3 illustrates the actual splitting of the two tissues.

**Table 1:** Statistics of SPF and non-SPF mouse transcriptome of the olfactory bulb and the nasal mucosa after one and two weeks mapping to July 2007 NCBI37/mm9 reference mouse genome.

Sample	raw read mean	raw read mean after quality control	filtered alignment mean (total unique mapping reads)
bulb, 1 week, SPF	28,741,596	28,005,180	22,695,537
bulb, 2 weeks, SPF	35,268,004	33,273,100	22,357,490
bulb, 1 week, non-SPF	47,813,480	47,022,748	36,626,425
bulb, 2 weeks, non-SPF	29,435,404	27,781,680	18,521,982
mucosa, 1 week, SPF	41,652,796	40,177,944	27,424,340
mucosa, 2 weeks, SPF	29,720,023	28,145,597	23,545,373
mucosa, 1 week, non-SPF	34,340,208	32,674,300	22,542,914
mucosa, 2 weeks, non-SPF	33,258,012	31,905,897	32,604,488
Mean	35,028,690	33,623,306	25,789,819

The samples of each tissue, divided by point of time and husbandry condition, were sequenced and the raw read mean was determined (mean≈ 35 mil reads). After quality control an average of 33.6 million reads per sample remained, of which 25.8 million were uniquely mapped to the reference genome.

### 2. Nasal mucosa shows strong immune response under non-SPF conditions

**Fig 4:**
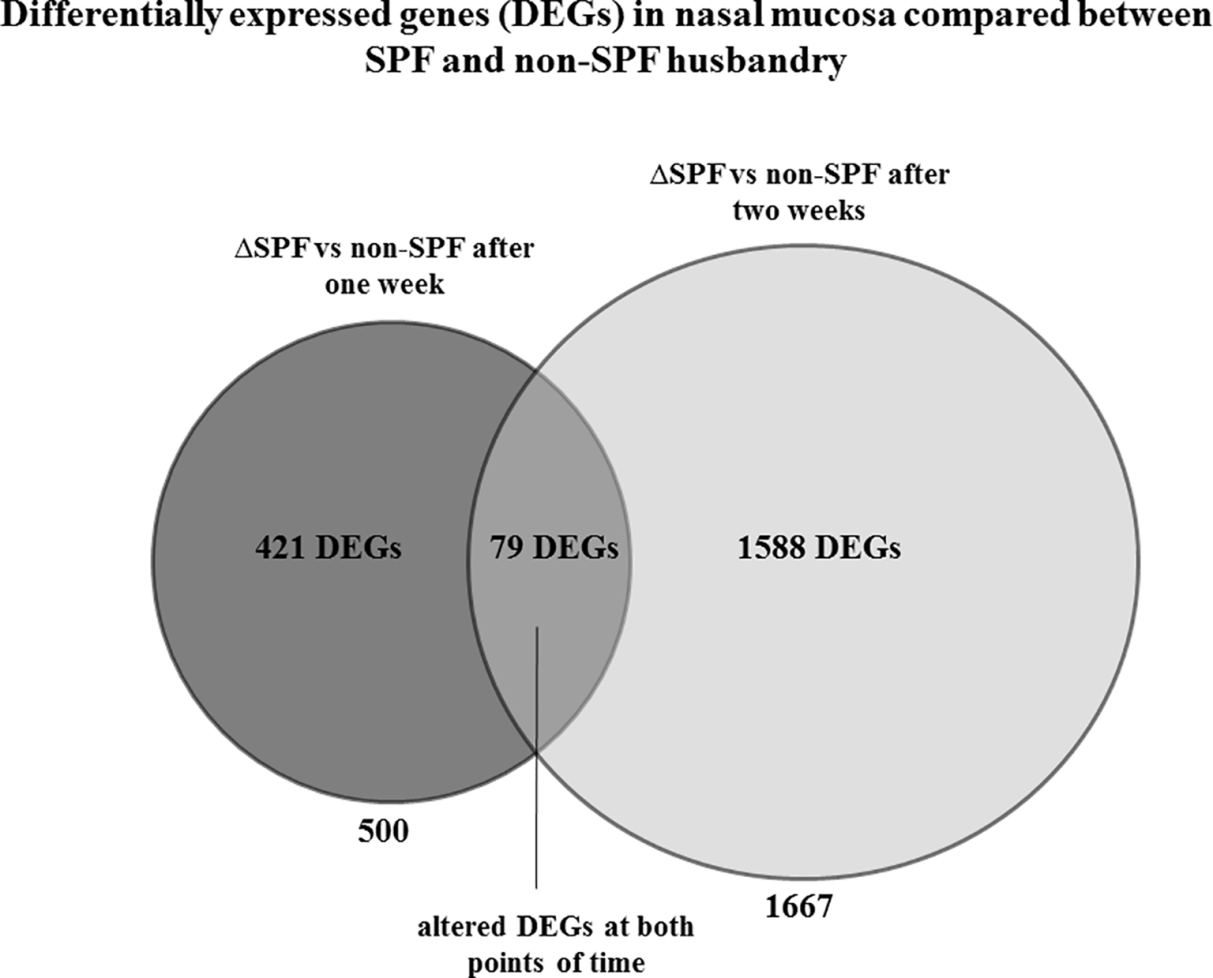
Non-SPF environment evokes a strong immune response in nasal mucosa. Comparison of nasal mucosa of both SPF and non-SPF conditions after one and two weeks showed a significant change of numerous transcripts. Overlap analysis revealed 421 DEGs after 7 days and 1,588 DEGs after 14 days.) The overlapping subset contained 79 DEGs.

Gene expression in nasal mucosa was compared between animals kept under SPF and non-SPF conditions at two points of time. After RNA deep sequencing, the subsets of DEGs resulting from SPF vs non-SPF environment were compared after one and two weeks, respectively. To this end, we selected from the 60 probes the 30 deriving from nasal mucosa, of which 15 came from SPF mice and 15 came from non-SPF mice. When compared between SPF and non-SPF husbandry the mucosal probes after one week showed 500 differentially expressed genes and the probes gained after two weeks resulted in 1.667 differentially expressed genes (DEGs) (Fig 4). Overlap analysis discloses 79 genes differentially expressed at both points of time (S1 Table). Further gene ontology analysis showed numerous immune-related GOs after one week as well as after two weeks (Table 2). These include leukocyte activation, regulation of lymphocyte activation and proliferation, as well as cytokine and chemokine activation.

**Table 2:** Gene ontology analysis of differentially expressed genes of nasal mucosa after one and two weeks when compared between SPF and non-SPF conditions revealed numerous immune-related GOs.

**Mucosa, 1 week**	**p-value**	**FDR**
GO:0008009 ~ chemokine activity	1,76E-04	2,52E-01
GO:0006955 ~ immune response	1,78E-04	2,99E-01
GO:0042379 ~ chemokine receptor binding	2,04E-04	2,92E-01
**Mucosa, 2 weeks**	**p-value**	**FDR**
GO:0006955~immune response	2,54E-08	4,61E-05
GO:0002694~regulation of leukocyte activation	8,71E-07	1,58E-03
GO:0045321~leukocyte activation	9,90E-07	1,80E-03
GO:0002252~immune effector process	4,05E-06	7,36E-03
GO:0050670~regulation of lymphocyte proliferation	9,65E-06	1,75E-02
GO:0002684~positive regulation of immune system process	1,04E-05	1,88E-02
GO:0070663~regulation of leukocyte proliferation	1,41E-05	2,56E-02
GO:0051249~regulation of lymphocyte activation	1,72E-05	3,13E-02
GO:0002520~immune system development	2,51E-05	4,55E-02
GO:0019955~cytokine binding	3,53E-05	5,65E-02
GO:0006952~defense response	5,19E-05	9,42E-02
GO:0004896~cytokine receptor activity	5,60E-05	8,96E-02
GO:0032944~regulation of mononuclear cell proliferation	9,65E-06	1,75E-02

Gene ontology analysis of differentially expressed genes of nasal mucosa when compared between SPF and non-SPF husbandry reveals in many significant (p-value<0.05;FDR<0.1) immune related GOs after both one and two weeks.

### 3. The olfactory bulb shows no immunological response

**Fig 5:**
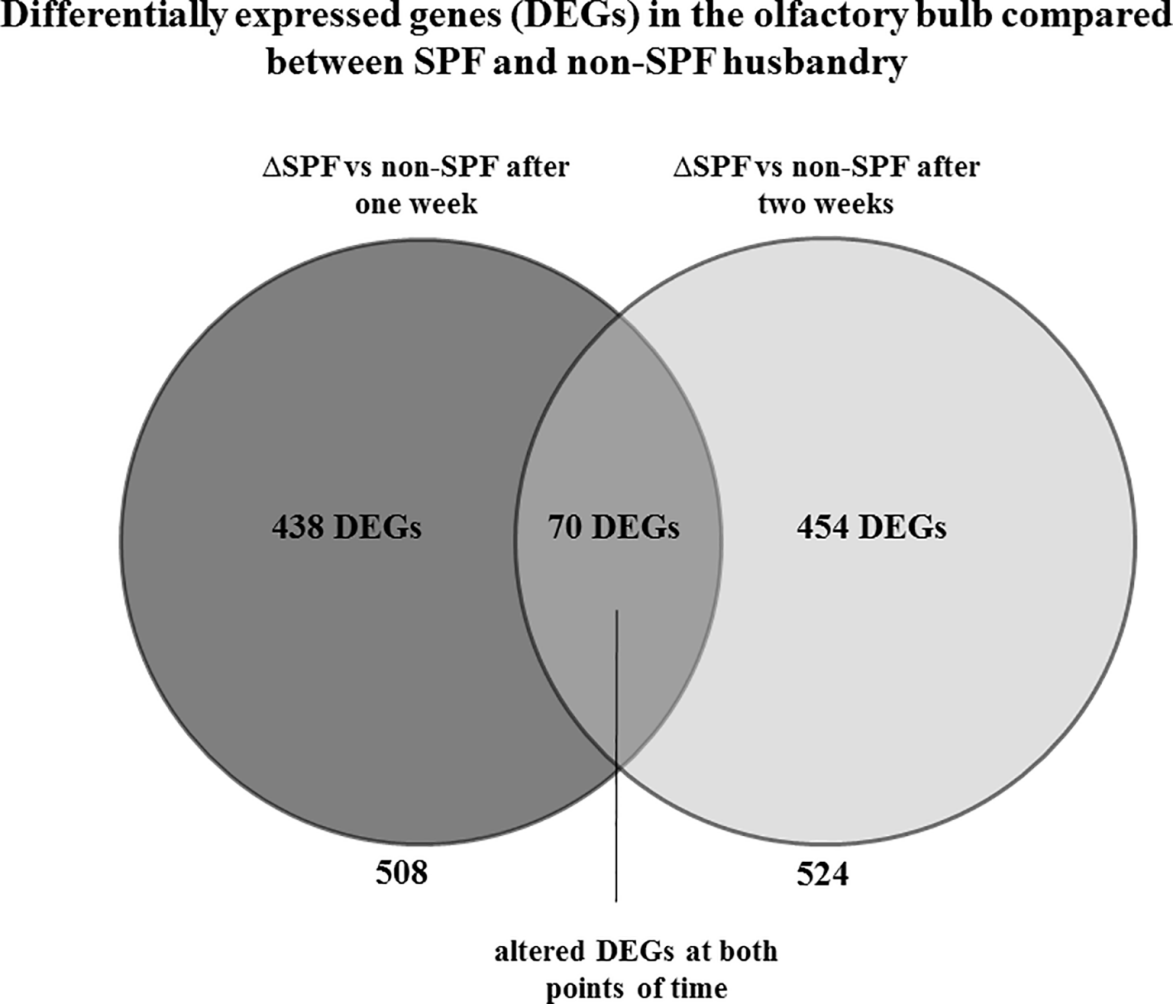
The olfactory bulb does not react immunologically. After both one week and two weeks there were significantly regulated genes in the olfactory bulb when comparing SPF to non-SPF conditions. To compare the regulated genes at each point of time, an overlap analysis was performed. 70 genes were regulated at both points of time (see S1 Table). 438 genes were regulated only after one week, 454 only after two weeks.

**Fig 6:**
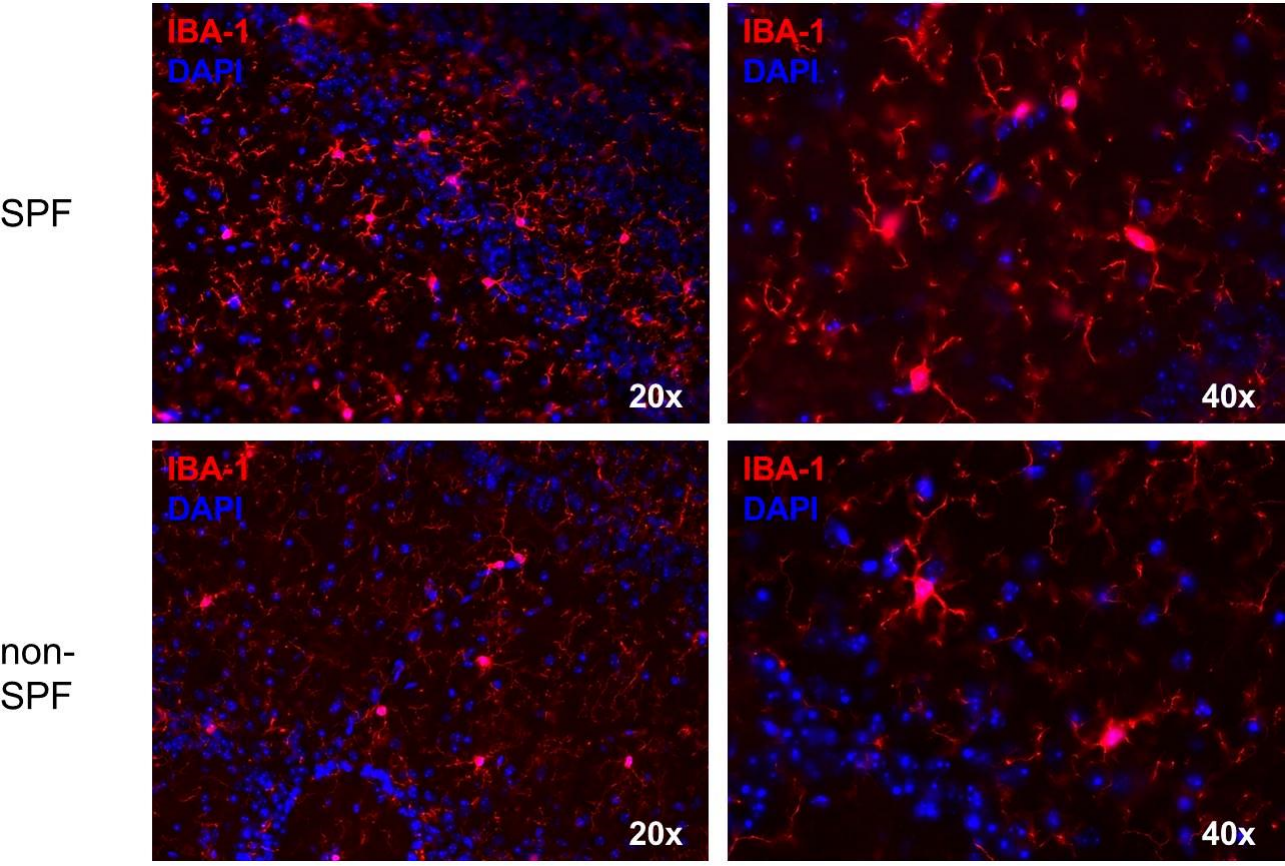
IBA-1 staining of olfactory bulb microglia of SPF and non-SPF mice show no morphological differences in terms of activation. The olfactory bulbs of mice kept under non-SPF conditions for five days and mice coming from a SPF environment were stained with the marker IBA-1 to examine if there are morphological signs for microglial activation in either group. As shown above no activation could be detected, as the microglia appears ramified in both conditions.

**Fig 7:**
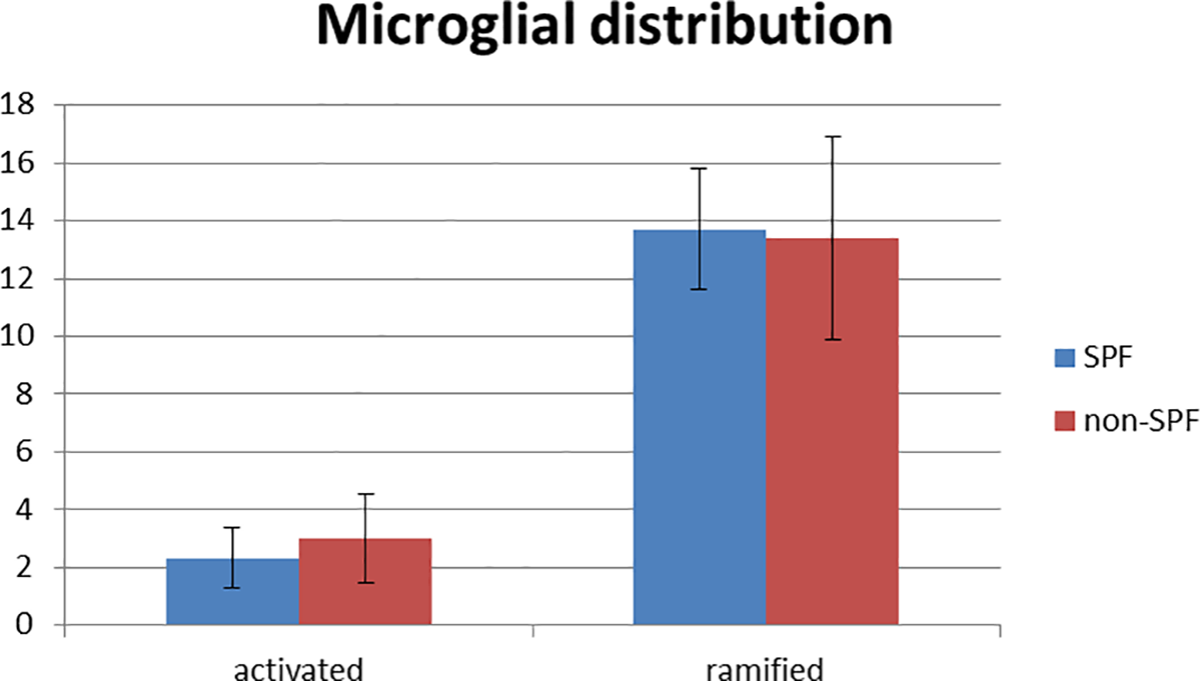
Quantification of activated and ramified microglia in the olfactory bulb under SPF and non-SPF conditions shows no difference in morphological microglial activation. The olfactory bulbs of mice kept under non-SPF conditions for 5 days and the olfactory bulbs of mice kept under SPF conditions were stained with IBA-1. Five pictures of each group were analyzed and the number of activated and ramified microglia was counted. No difference in microglial activation between SPF and non-SPF conditions was found.

To investigate if the olfactory bulb senses commensalism of the adjacent mucosa, we kept a group of 15 mice in an SPF breeding facility for comparison to 15 animals housed in a “dirty”, non-SPF facility. There were 508 genes differently expressed significantly after one week, whereas 524 DEGs were identified after two weeks when compared between SPF and non-SPF husbandry. To identify genes which were differently expressed at both points of time, we conducted an overlap analysis (Fig 5) which resulted in 70 DEGs (S2 Table). 438 and 454 genes were differentially expressed only after one week and two weeks, respectively. Gene Ontology (GO) analysis was performed to determine groups of differentially expressed genes for the three subsets (differentially expressed only after one week, differentially expressed only after two weeks, differentially expressed at both points of time). Gene ontology analysis revealed no significant (p<0.05; FDR>0.1) GO for differentially changed transcripts after one week and for the genes overlapping at both, one and two weeks (data not shown). One single GO (GO: *extracellular matrix*) was identified for the two weeks data. Our data show that there are indeed differences in the transcriptional response of the OB due to the different environments, but this does not affect immunological candidates and pathways. Since these results were unexpected, we specifically analysed the expression of genes required for immune responses such as antimicrobial peptides, interleukins, and interferons. However, we found no significant differently expressed immune-related genes (S3 Table). Furthermore we performed MHC II/IBA-1 double stainings to examine if the immunological answer in the olfactory bulb takes place earlier than one week (after one, three and five days, respectively) of non-SPF environment. Eventually, no MHC II was detectable (data not shown). In addition Fig 6 reveals no morphological differences in microglia regarding their activation state in olfactory bulbs of mice with an SPF and non-SPF background, respectively. For further quantification we analyzed five pictures of olfactory bulbs from animals kept under SPF conditions and animals kept under non-SPF conditions for five days in order to determine the rate of activated vs. ramified microglia. Fig 7 shows no difference of microglia.activation between SPF and non-SPF husbandry. Hence we have neither histological nor transcriptional evidence to support our hypothesis that commensalism due to non-SPF conditions induces immunological defence mechanisms in the olfactory bulb.

## Discussion

In this study we kept two subgroups of animals in an SPF and non-SPF environment, respectively. We then isolated the nasal mucosa and olfactory bulb each to study the immunological response within these tissues by using next generation sequencing of transcripts. It should be noted that changes in mRNA levels can be either caused by regulation of gene expression or changes in cell composition which both would be indicative of an immune response. We regard it as our major finding that—against our hypothesis—expression of immune-related genes was not changed in the olfactory bulb although the FELASA protocol notes the presence of MHV and *Syphacia species* in sentinel mice of the animal facility. Although *syphacia* is not known for infections of the central nervous system (CNS), at least some MHV strains are well known neurotropic viruses [19], which can induce demyelinating disease when experimentally infected. We have previously used VSV-eGFP to study the transportation of virus from nose to bulb [8] and MHV-N to study immune defense mechanisms of the olfactory bulb [20]. In the current study,the husbandry conditions were distinctly different in terms of hygiene, accessibility and stable environment. We therefore considered it likely that at least genes coding for IFN-γ, TNF-α or IL-1 are induced e.g. by Toll-like receptor or NF-κB signalling. This was, however, not the case (see S3 Table). Thus, in contrast to direct intranasal or ocular infection where cytokines such as IL-1, IL-6, IFN-ß or TNF-α were strongly altered [21–24], stimulation in our environment of interest apparently was not sufficient to provoke a similar response. This environment, however, caused massive changes in the nasal mucosa of the animals kept under non-SPF circumstances. The significantly changed gene ontology groups include chemokine activity and binding, leukocyte activation and proliferation as well as more general terms such as defense response, immune response and immune effector process. Obviously, our non-SPF environment of interest is adequate to trigger a broad immune response in the nasal mucosa.These findings suggest that in a non-SPF environment, the nasal mucosa provides a sufficient border to prevent pathogens from progressing further down the olfactory route. Indeed, microglia which strongly upregulate MHC-II expression upon intranasal infection with vesicular stomatitis virus (VSV) (unpublished observation) remained immune negative after one, three, and five days under non-SPF conditions, and also did not exhibit morphological signs of activation. A recent study showed that the immune system of laboratory mice kept under SPF circumstances massively varies from the one of pet shop mice. The former were much more similar to new born babies than to adults raising concerns against the common extrapolation of respective data to the human situation [6]. Furthermore, a study of Reese et al. (2016) [7] shows that the immunological response to vaccines differs massively in terms of gene expression, particularly cytokine expression, and quantity of antibodies between mice kept in an immunological unchallenging barrier and mice which were previously co-infected with common pathogenes such as helminths and herpesviruses. These recent observations indicate the importance of experiments involving immunological challenging environments for immunological research. We used such adjustement to a more realistic environment to capture the transcriptomic response in the olfactory bulb and nasal mucosa involved in homeostatic defense. Our data show that the mucosa appears to provide a sufficient response to environmental pathogens as the challenge of open housing does not induce an immunological reaction at the transcriptional level in the olfabctory bulb. The list of genes induced upon moving from SPF to a more challenging environment may include candidates who’s mutations may render risk for the development of meningitis.

## Supporting information

S1 Table Significant overlapping of differentially expressed transcripts at both points of time in the nasal mucosa when compared between SPF and non-SPF. Overlap analysis revealed in 79 significant differentially expressed genes of the nasal mucosa when compared between SPF and non-SPF both after one week and two weeks.
(DOCX)

S2 Table Significant overlapping of differentially expressed transcripts at both points of time in the olfactory bulb when compared between SPF and non-SPF. Overlap analysis showed 70 significant differentially expressed genes of olfactory bulb when compared between SPF and non-SPF both after one week and two weeks.
(DOCX)

S3 Table Differential expression of selected immunological genes in the olfactory bulb after 1 week when compared between SPF and non-SPF environment. To further investigate if the olfactory bulb reacts immunologically when challenged with an open environment we searched for expression of typical immunological genes after one week of non-SPF husbandry. Our data show no significance in expression of these genes in the olfactory bulb when compared between SPF and non-SPF environment.
(DOCX)
