# Supplementary material for: Open housing drives the expression of immune response genes in the nasal mucosa, but not the olfactory bulb
Source: PLoS One. 2017 Oct 27;12(10):e0187192. doi: 10.1371/journal.pone.0187192 (PMC5659768; doi:10.1371/journal.pone.0187192)
Supplement: S1 Table — Overlap analysis revealed in 79 significant differentially expressed genes of the nasal mucosa when compared between SPF and non-SPF both after one week and two weeks. (DOCX) [file pone.0187192.s001.docx]

S1 Table

| external_gene_id | ensembl_gene_id | Description |  |  |
| --- | --- | --- | --- | --- |
| Tat | ENSMUSG00000001670 | tyrosine aminotransferase | | |
| Angptl4 | ENSMUSG00000002289 | angiopoietin-like 4 | |  |
| Ly9 | ENSMUSG00000004707 | lymphocyte antigen 9 | |  |
| Trh | ENSMUSG00000005892 | thyrotropin releasing hormone | | |
| Derl3 | ENSMUSG00000009092 | Der1-like domain family, member 3 | | |
| Tnfrsf13b | ENSMUSG00000010142 | tumor necrosis factor receptor superfamily, member 13b | | |
| Cybb | ENSMUSG00000015340 | cytochrome b-245, beta polypeptide | | |
| Tubb1 | ENSMUSG00000016255 | tubulin, beta 1 class VI | |  |
| Lims1 | ENSMUSG00000019920 | LIM and senescent cell antigen-like domains 1 | | |
| Nampt | ENSMUSG00000020572 | nicotinamide phosphoribosyltransferase | | |
| Apob | ENSMUSG00000020609 | apolipoprotein B | |  |
| 9030617O03Rik | ENSMUSG00000021185 | RIKEN cDNA 9030617O03 gene | | |
| Mctp1 | ENSMUSG00000021596 | multiple C2 domains, transmembrane 1 | | |
| Cyp2d26 | ENSMUSG00000022445 | cytochrome P450, family 2, subfamily d, polypeptide 26 | | |
| Kng1 | ENSMUSG00000022875 | kininogen 1 | |  |
| Cbr3 | ENSMUSG00000022947 | carbonyl reductase 3 | |  |
| Setd4 | ENSMUSG00000022948 | SET domain containing 4 | |  |
| Tnfrsf12a | ENSMUSG00000023905 | tumor necrosis factor receptor superfamily, member 12a | | |
| Megf10 | ENSMUSG00000024593 | multiple EGF-like-domains 10 | | |
| Sorbs1 | ENSMUSG00000025006 | sorbin and SH3 domain containing 1 | | |
| Pfkfb4 | ENSMUSG00000025648 | 6-phosphofructo-2-kinase/fructose-2,6-biphosphatase 4 | | |
| Tnni1 | ENSMUSG00000026418 | troponin I, skeletal, slow 1 | | |
| Lrrn2 | ENSMUSG00000026443 | leucine rich repeat protein 2, neuronal | | |
| Gad2 | ENSMUSG00000026787 | glutamic acid decarboxylase 2 | | |
| Itga4 | ENSMUSG00000027009 | integrin alpha 4 | |  |
| Slc7a11 | ENSMUSG00000027737 | solute carrier fam. 7 (cation. amino acid transp., y+ syst.), mem 11 | | |
| Mme | ENSMUSG00000027820 | membrane metallo endopeptidase | | |
| Aldob | ENSMUSG00000028307 | aldolase B, fructose-bisphosphate | | |
| Ambp | ENSMUSG00000028356 | alpha 1 microglobulin/bikunin | | |
| Ptplad2 | ENSMUSG00000028497 | protein tyrosine phosphatase-like A domain containing 2 | | |
| Sult1e1 | ENSMUSG00000029272 | sulfotransferase family 1E, member 1 | | |
| Alb | ENSMUSG00000029368 | albumin |  |  |
| Qprt | ENSMUSG00000030674 | quinolinate phosphoribosyltransferase | | |
| Zp2 | ENSMUSG00000030911 | zona pellucida glycoprotein 2 | | |
| Btg4 | ENSMUSG00000032056 | B cell translocation gene 4 | | |
| Apoa1 | ENSMUSG00000032083 | apolipoprotein A-I | |  |
| Thsd4 | ENSMUSG00000032289 | thrombospondin, type I, domain containing 4 | | |
| Gclc | ENSMUSG00000032350 | glutamate-cysteine ligase, catalytic subunit | | |
| Gas7 | ENSMUSG00000033066 | growth arrest specific 7 | |  |
| St18 | ENSMUSG00000033740 | suppression of tumorigenicity 18 | | |
| Fgb | ENSMUSG00000033831 | fibrinogen beta chain | |  |
| Neurod1 | ENSMUSG00000034701 | neurogenic differentiation 1 | | |
| Gc | ENSMUSG00000035540 | group specific component | | |
| Avp | ENSMUSG00000037727 | arginine vasopressin | |  |
| Atf7ip2 | ENSMUSG00000039200 | activating transcription factor 7 interacting protein 2 | | |
| Bach2 | ENSMUSG00000040270 | BTB and CNC homology 2 | | |
| Ninj2 | ENSMUSG00000041377 | ninjurin 2 |  |  |
| Specc1 | ENSMUSG00000042331 | sperm antigen with calponin homology and coiled-coil domains 1 | | |
| Grap2 | ENSMUSG00000042351 | GRB2-related adaptor protein 2 | | |
| 9930012K11Rik | ENSMUSG00000044551 | RIKEN cDNA 9930012K11 gene | | |
| Ttc39d | ENSMUSG00000046196 | tetratricopeptide repeat domain 39D | | |
| Gpr156 | ENSMUSG00000046961 | G protein-coupled receptor 156 | | |
| Sox6 | ENSMUSG00000051910 | SRY-box containing gene 6 | | |
| Fabp1 | ENSMUSG00000054422 | fatty acid binding protein 1, liver | | |
| Cyp3a11 | ENSMUSG00000056035 | cytochrome P450, family 3, subfamily a, polypeptide 11 | | |
| AI504432 | ENSMUSG00000056145 | expressed sequence AI504432 | | |
| Nxf3 | ENSMUSG00000057000 | nuclear RNA export factor 3 | | |
| Serpina3k | ENSMUSG00000058207 | serine (or cysteine) peptidase inhibitor, clade A, member 3K | | |
| Caln1 | ENSMUSG00000060371 | calneuron 1 | |  |
| Ttr | ENSMUSG00000061808 | transthyretin | |  |
| Olfr99 | ENSMUSG00000061972 | olfactory receptor 99 | |  |
| Cyp26b1 | ENSMUSG00000063415 | cytochrome P450, family 26, subfamily b, polypeptide 1 | | |
| Mup3 | ENSMUSG00000066154 | major urinary protein 3 | |  |
| Klk1b5 | ENSMUSG00000066512 | kallikrein 1-related peptidase b5 | | |
| Cyp2d9 | ENSMUSG00000068086 | cytochrome P450, family 2, subfamily d, polypeptide 9 | | |
| Serpina1b | ENSMUSG00000071178 | serine (or cysteine) preptidase inhibitor, clade A, member 1B | | |
| Plac9b | ENSMUSG00000072674 | placenta specific 9b | |  |
| Serpina1e | ENSMUSG00000072849 | serine (or cysteine) peptidase inhibitor, clade A, member 1E | | |
| Mup11 | ENSMUSG00000073834 | major urinary protein 11 | |  |
| Gm1966 | ENSMUSG00000073902 | predicted gene 1966 | |  |
| Ceacam1 | ENSMUSG00000074272 | carcinoembryonic antigen-related cell adhesion molecule 1 | | |
| Serpina1c | ENSMUSG00000079015 | serine (or cysteine) peptidase inhibitor, clade A, member 1C | | |
| Nhsl2 | ENSMUSG00000079481 | NHS-like 2 |  |  |
| Mettl21b | ENSMUSG00000080115 | methyltransferase like 21B | | |
| Mir22hg | ENSMUSG00000085148 | Mir22 host gene (non-protein coding) | | |
| Snhg3 | ENSMUSG00000085241 | small nucleolar RNA host gene (non-protein coding) 3 | | |
| B430119L08Rik | ENSMUSG00000086432 | RIKEN cDNA B430119L08 gene | | |
| NA | ENSMUSG00000074860 | NA |  |  |
| NA | ENSMUSG00000089681 | NA |  |  |
