# Supplementary material for: Open housing drives the expression of immune response genes in the nasal mucosa, but not the olfactory bulb
Source: PLoS One. 2017 Oct 27;12(10):e0187192. doi: 10.1371/journal.pone.0187192 (PMC5659768; doi:10.1371/journal.pone.0187192)
Supplement: S2 Table — Overlap analysis showed 70 significant differentially expressed genes of olfactory bulb when compared between SPF and non-SPF both after one week and two weeks. (DOCX) [file pone.0187192.s002.docx]

S2 Table

| external_gene_id | ensembl_gene_id | description |  |  |  |
| --- | --- | --- | --- | --- | --- |
| Akap8l | ENSMUSG00000002625 | A kinase (PRKA) anchor protein 8-like | | | |
| Calr | ENSMUSG00000003814 | calreticulin | |  |  |
| Zmynd10 | ENSMUSG00000010044 | zinc finger, MYND domain containing 10 | | | |
| Odc1 | ENSMUSG00000011179 | ornithine decarboxylase, structural 1 | | |  |
| Pltp | ENSMUSG00000017754 | phospholipid transfer protein | | |  |
| Plagl1 | ENSMUSG00000019817 | pleiomorphic adenoma gene-like 1 | | |  |
| Ddc | ENSMUSG00000020182 | dopa decarboxylase | |  |  |
| Pdia6 | ENSMUSG00000020571 | protein disulfide isomerase associated 6 | | | |
| Scgn | ENSMUSG00000021337 | secretagogin, EF-hand calcium binding protein | | | |
| Pdzd2 | ENSMUSG00000022197 | PDZ domain containing 2 | | |  |
| Dlg1 | ENSMUSG00000022770 | discs, large homolog 1 (Drosophila) | | |  |
| Pde10a | ENSMUSG00000023868 | phosphodiesterase 10A | |  |  |
| Xdh | ENSMUSG00000024066 | xanthine dehydrogenase | | |  |
| Ldlrad4 | ENSMUSG00000024544 | low density lipoprotein receptor class A domain containing 4 | | | |
| Banp | ENSMUSG00000025316 | BTG3 associated nuclear protein | | |  |
| Cpeb1 | ENSMUSG00000025586 | cytoplasmic polyadenylation element binding protein 1 | | | |
| Homer2 | ENSMUSG00000025813 | homer homolog 2 (Drosophila) | | |  |
| Sox17 | ENSMUSG00000025902 | SRY-box containing gene 17 | | |  |
| Rgs5 | ENSMUSG00000026678 | regulator of G-protein signaling 5 | | |  |
| Lcn4 | ENSMUSG00000026919 | lipocalin 4 |  |  |  |
| Ppm1l | ENSMUSG00000027784 | protein phosphatase 1 (formerly 2C)-like | | | |
| Lef1 | ENSMUSG00000027985 | lymphoid enhancer binding factor 1 | | |  |
| Ccdc109b | ENSMUSG00000027994 | coiled-coil domain containing 109B | | |  |
| Pappa | ENSMUSG00000028370 | pregnancy-associated plasma protein A | | | |
| Slc2a1 | ENSMUSG00000028645 | solute carrier family 2 (facilitated glucose transporter), member 1 | | | |
| Sorcs2 | ENSMUSG00000029093 | sortilin-related VPS10 domain containing receptor 2 | | | |
| Abcg2 | ENSMUSG00000029802 | ATP-binding cassette, sub-family G (WHITE), member 2 | | | |
| Gkn3 | ENSMUSG00000030048 | gastrokine 3 | |  |  |
| Gxylt2 | ENSMUSG00000030074 | glucoside xylosyltransferase 2 | | |  |
| Slco1a4 | ENSMUSG00000030237 | solute carrier organic anion transporter family, member 1a4 | | | |
| Prmt3 | ENSMUSG00000030505 | protein arginine N-methyltransferase 3 | | | |
| D430042O09Rik | ENSMUSG00000032743 | RIKEN cDNA D430042O09 gene | | |  |
| Cdo1 | ENSMUSG00000033022 | cysteine dioxygenase 1, cytosolic | | |  |
| Tie1 | ENSMUSG00000033191 | tyrosine kinase with immunoglobulin-like and EGF-like domains 1 | | | |
| Bzrap1 | ENSMUSG00000034156 | benzodiazepine receptor associated protein 1 | | | |
| Shq1 | ENSMUSG00000035378 | SHQ1 homolog (S. cerevisiae) | | |  |
| Galnt18 | ENSMUSG00000038296 | UDP-N-acetyl-alpha-D-galactosamine | | | |
| Arntl2 | ENSMUSG00000040187 | aryl hydrocarbon receptor nuclear translocator-like 2 | | | |
| Cchcr1 | ENSMUSG00000040312 | coiled-coil alpha-helical rod protein 1 | | | |
| Ltbp4 | ENSMUSG00000040488 | latent transforming growth factor beta binding protein 4 | | | |
| Abcb1a | ENSMUSG00000040584 | ATP-binding cassette, sub-family B (MDR/TAP), member 1A | | | |
| Cyyr1 | ENSMUSG00000041134 | cysteine and tyrosine-rich protein 1 | | |  |
| Lhfpl1 | ENSMUSG00000041700 | lipoma HMGIC fusion partner-like 1 | | |  |
| Pde3a | ENSMUSG00000041741 | phosphodiesterase 3A, cGMP inhibited | | | |
| S100a10 | ENSMUSG00000041959 | S100 calcium binding protein A10 (calpactin) | | | |
| Mustn1 | ENSMUSG00000042485 | musculoskeletal, embryonic nuclear protein 1 | | | |
| Fam166b | ENSMUSG00000042788 | family with sequence similarity 166, member B | | | |
| Hs3st5 | ENSMUSG00000044499 | heparan sulfate (glucosamine) 3-O-sulfotransferase 5 | | | |
| Dlgap2 | ENSMUSG00000047495 | discs, large (Drosophila) homolog-associated protein 2 | | | |
| Amigo2 | ENSMUSG00000048218 | adhesion molecule with Ig like domain 2 | | | |
| Dip2c | ENSMUSG00000048264 | DIP2 disco-interacting protein 2 homolog C (Drosophila) | | | |
| Crh | ENSMUSG00000049796 | corticotropin releasing hormone | | |  |
| Scg2 | ENSMUSG00000050711 | secretogranin II | |  |  |
| Sv2c | ENSMUSG00000051111 | synaptic vesicle glycoprotein 2c | | |  |
| Bbs12 | ENSMUSG00000051444 | Bardet-Biedl syndrome 12 (human) | | |  |
| Slc8a1 | ENSMUSG00000054640 | solute carrier family 8 (sodium/calcium exchanger), member1 | | | |
| Gpr116 | ENSMUSG00000056492 | G protein-coupled receptor 116 | | |  |
| Cep110 | ENSMUSG00000057110 | centrosomal protein 110 | | |  |
| Ankrd29 | ENSMUSG00000057766 | ankyrin repeat domain 29 | | |  |
| Tnip2 | ENSMUSG00000059866 | TNFAIP3 interacting protein 2 | | |  |
| Maml3 | ENSMUSG00000061143 | mastermind like 3 (Drosophila) | | |  |
| Obp2a | ENSMUSG00000062061 | odorant binding protein 2A | | |  |
| Hmcn1 | ENSMUSG00000066842 | hemicentin 1 | |  |  |
| Armc2 | ENSMUSG00000071324 | armadillo repeat containing 2 | | |  |
| Mcc | ENSMUSG00000071856 | mutated in colorectal cancers | | |  |
| E530001K10Rik | ENSMUSG00000075020 | RIKEN cDNA E530001K10 gene | | |  |
| Ly6c1 | ENSMUSG00000079018 | lymphocyte antigen 6 complex, locus C1 | | | |
| Obp2b | ENSMUSG00000079539 | odorant binding protein 2B | | |  |
| NA | ENSMUSG00000078437 | NA |  |  |  |
| NA | ENSMUSG00000086242 | NA |  |  |  |
