# Supplementary material for: Open housing drives the expression of immune response genes in the nasal mucosa, but not the olfactory bulb
Source: PLoS One. 2017 Oct 27;12(10):e0187192. doi: 10.1371/journal.pone.0187192 (PMC5659768; doi:10.1371/journal.pone.0187192)
Supplement: S3 Table — To further investigate if the olfactory bulb reacts immunologically when challenged with an open environment we searched for expression of typical immunological genes after one week of non-SPF husbandry. Our data show no significance in expression of these genes in the olfactory bulb when compared between SPF and non-SPF environment. (DOCX) [file pone.0187192.s003.docx]

S3 Table

| ensembl_gene_id | baseMean | log2FoldChange | pval | description |  |  |  |
| --- | --- | --- | --- | --- | --- | --- | --- |
| ENSMUSG00000074681 | 6.175 | -0.211 | 0.640 | defensin beta 23 | |  |  |
| ENSMUSG00000054763 | 3.171 | -0.177 | 0.640 | defensin beta 42 | |  |  |
| ENSMUSG00000069515 | 3.532 | -0.098 | 0.965 | lysozyme 1 |  |  |  |
| ENSMUSG00000069516 | 862.260 | 0.058 | 0.838 | lysozyme 2 |  |  |  |
| ENSMUSG00000038357 | 177.376 | -1.325 | 0.357 | cathelicidin antimicrobial peptide | | |  |
| ENSMUSG00000036216 | 6.224 | -0.322 | 0.840 | liver-expressed antimicrobial peptide 2 | | | |
| ENSMUSG00000017652 | 116.088 | -0.686 | 0.205 | CD40 antigen | |  |  |
| ENSMUSG00000023274 | 48.189 | -1.377 | 0.150 | CD4 antigen |  |  |  |
| ENSMUSG00000022901 | 349.610 | -0.346 | 0.148 | CD86 antigen | |  |  |
| ENSMUSG00000030786 | 7302.453 | 0.024 | 0.933 | integrin alpha M (CD11b) | |  |  |
| ENSMUSG00000030789 | 60.468 | -0.357 | 0.275 | integrin alpha X (CD11c) | |  |  |
| ENSMUSG00000067149 | 39.983 | 0.250 | 0.790 | immunoglobulin joining chain | | |  |
| ENSMUSG00000020009 | 1970.046 | 0.023 | 0.991 | interferon gamma receptor 1 | | |  |
| ENSMUSG00000022965 | 1373.013 | 0.234 | 0.394 | interferon gamma receptor 2 | | |  |
| ENSMUSG00000022967 | 6536.058 | 0.066 | 0.797 | interferon (alpha and beta) receptor 1 | | | |
| ENSMUSG00000022971 | 4703.854 | 0.095 | 0.669 | interferon (alpha and beta) receptor 2 | | | |
| ENSMUSG00000042993 | 14.048 | -0.336 | 0.426 | interferon kappa | |  |  |
| ENSMUSG00000027399 | 39.672 | 0.251 | 0.959 | interleukin 1 alpha | |  |  |
| ENSMUSG00000027398 | 19.821 | -1.256 | 0.124 | interleukin 1 beta | |  |  |
| ENSMUSG00000000869 | 180.607 | 0.101 | 0.697 | interleukin 4 |  |  |  |
| ENSMUSG00000025746 | 3.614 | -1.823 | 0.273 | interleukin 6 |  |  |  |
| ENSMUSG00000027776 | 99.115 | 0.248 | 0.690 | interleukin 12a | |  |  |
| ENSMUSG00000046108 | 3.713 | -0.328 | 0.880 | interleukin 17C | |  |  |
| ENSMUSG00000025383 | 9.934 | -0.661 | 0.525 | interleukin 23, alpha subunit p19 | | |  |
| ENSMUSG00000024778 | 126.259 | 0.364 | 0.585 | Fas (TNF receptor superfamily member 6) | | | |
| ENSMUSG00000000817 | 13.972 | -0.501 | 0.246 | Fas ligand (TNF superfamily, member 6) | | | |
| ENSMUSG00000036594 | 689.167 | -0.075 | 0.418 | histocompatibility 2, class II antigen A, alpha | | | |
| ENSMUSG00000001864 | 639.009 | -0.002 | 0.801 | allograft inflammatory factor 1-like (IBA-1) | | | |
| ENSMUSG00000039005 | 223.734 | 0.818 | 1.000 | toll-like receptor 4 | |  |  |
| ENSMUSG00000027995 | 65.175 | 0.887 | 1.000 | toll-like receptor 2 | |  |  |
| ENSMUSG00000021025 | 1149.159 | 0.532 | 1.000 | nuclear factor of kappa light polypeptide | | | |
|  |  |  |  | gene enhancer in B cells inhibitor, alpha | | | |
| ENSMUSG00000030595 | 2559.305 | 0.885 | 1.000 | nuclear factor of kappa light polypeptide | | | |
|  |  |  |  | gene enhancer in B cells inhibitor, beta | | | |
| ENSMUSG00000032041 | 72.393 | 0.408 | 1.000 | toll-interleukin 1 receptor (TIR) | | |  |
|  |  |  |  | domain-containing adaptor protein | | |  |
| ENSMUSG00000026656 | 1528.858 | 0.746 | 1.000 | Fc receptor, IgG, low affinity IIb | | |  |
| ENSMUSG00000021484 | 2100.243 | 0.743 | 1.000 | lectin, mannose-binding 2 | |  |  |
| ENSMUSG00000005981 | 8492.069 | 0.796 | 1.000 | TNF receptor-associated protein 1 | | |  |
| ENSMUSG00000017615 | 3506.616 | 0.990 | 1.000 | tumor necrosis factor, alpha-induced | | | |
|  |  |  |  | protein 1 (endothelial) | |  |  |
| ENSMUSG00000032089 | 264.919 | 0.454 | 1.000 | interleukin 10 receptor, alpha | | |  |
| ENSMUSG00000037523 | 592.094 | 0.993 | 1.000 | mitochondrial antiviral signaling protein | | | |
| ENSMUSG00000038058 | 340.997 | 0.223 | 1.000 | nucleotide-binding oligomerization | | |  |
|  |  |  |  | domain containing 1 | |  |  |
